# Occupational allergic contact urticaria to tropomyosin from squid 

**DOI:** 10.5414/ALX02121E

**Published:** 2020-12-08

**Authors:** Daniel Wilfinger, Annette Kuehn, Szandra Takacs, Evgenia Galli-Novak, Barbara Machan, Werner Aberer

**Affiliations:** 1 Department of Occupational Diseases and Occupational Medicine, AUVA Rehabilitation Clinic Tobelbad, Tobelbad, Austria,; 2 Department of Infection and Immunity, Luxembourg Institute of Health, Luxemburg, and; 3 Department of Dermatology and Venerology, Medical University Graz, Graz, Austria

**Keywords:** contact urticaria, squid, tropomyosin, IgE antibody, occupational disease

## Abstract

A cook’s mate working in an Austrian restaurant reported acutely occurring urticarial skin lesions after processing and cooking squid. The prick-to-prick test with squid showed a ++ positive urticarial reaction. Elevated specific IgE antibody levels to squid, shrimp, and house dust mites as well as to tropomyosin from shrimp and house dust mite could be detected in the ImmunoCAP. By means of immunoblot and ELISA, a reaction to squid extract as well as increased IgE antibody levels to squid and tropomyosin from squid could be detected. The patient was diagnosed with a clinically and occupationally relevant type I allergy to squid with cross-reaction to tropomyosin of other invertebrates and therefore recognized as an occupational disease.

**German version published in Allergologie, Vol. 43, No. 1/2020, pp. 3-9**

## Introduction 

In 2015, the Austrian Workers’ Compensation Board (AUVA) in cooperation with the Medical University of Graz introduced a prevention model for patients with occupational skin diseases based on the German model. If occupational skin diseases are suspected, dermatologic workup and therapy as well as secondary and tertiary prevention measures are provided in defined institutions. The dermatological workup of the presented case report was performed within the framework of this concept [1]. 

Food allergies affect ~ 3.5 – 4% of the population worldwide. Specific IgE antibodies against proteins naturally occurring in food cause allergic immediate-type reactions [2]. Fish and seafood are among the most common triggers of allergic and anaphylactic reactions worldwide [2, 3]. While parvalbumins are the most important allergen group for fish allergy, the main allergen for seafood allergy is tropomyosin [4, 5]. Tropomyosin is a muscle protein of invertebrates and is found not only in various seafood species but also, with a high degree of similarity, in dust mites and cockroaches. In contrast to tropomyosin from invertebrates, tropomyosin from vertebrates exerts almost no sensitizing potential [6, 7]. In fish, tropomyosin has only been described as an allergen for tilapia (Oreochromis mossambicus) [8]. For this reason, food allergy to seafood can lead to cross-reactions with other seafood species and other invertebrates such as dust mites, but cross-reactions to fish do usually not occur. Tropomyosin has been particularly well studied as an allergen from crustaceans, and was first described as a shrimp allergen in 1981 [9]. Since then, various tropomyosins from various invertebrates such as squid, mussels, snails, mites, cockroaches, and mealworms have been described [10, 11, 12, 13]. 

In addition to tropomyosin, other seafood allergens such as AK (arginine kinase), MLC (myosin light chain), SCP (sarcoplasmic calcium-binding protein), troponin, TIM (triose phosphate isomerase), and paramyosin have been published in recent years [8, 10, 14]. However, due to its function in muscle, tropomyosin is present in significantly higher amounts than the other allergens mentioned [14]. 

In the following, a case of an occupational type I allergy to tropomyosin from seafood will be reported. 

## Case report 

The female patient, who was 32 years old at the time of initial presentation, presented after reporting a suspected (allergy-related) occupational disease of the skin and lungs. 

The patient worked as a cook’s mate from 2006 to 2015 and reported suffering from itchy and reddened skin lesions on both hands between 2006 and 2010. The patient assumed the processing of raw squid to be the main cause. She also stated that she suffered from respiratory distress when cooking squid. The patient did not know which squid species was involved. 

She reported the immediate appearance of itchy erythema on both hands upon contact with raw squid (when washing and cleaning the seafood). Furthermore, she described that these skin lesions healed quickly and recurred with renewed contact with squid. Discomfort after contact with other foods, especially other seafood and fish were negated. Furthermore, the patient stated that she had not suffered from skin changes either before or since changing this job. 

The occupational investigation confirmed direct contact with squid while working as a cook’s mate in an Austrian restaurant from 2006 to 2010. After changing her job in 2010, there was no further exposure to squid. The patient did not use personal protective equipment (gloves, skin protection creams) during her employment in the kitchen. Additional skin exposures were reported for wet work of more than 2 hours daily and frequent washing of hands with soap and hand disinfection. 

The patient did not consult a physician in the period from 2006 to 2010, so there were no objective clinical findings from this period. At the time of the current study, the skin lesions had already occurred more than 7 years ago, so it was not possible to objectify them here either. The Erlangen atopy score was 8 points and thus showed no clear evidence of atopic diathesis. 

## Laboratory examination methods 

ImmunoCAP was used to analyze total IgE and specific IgE antibodies against squid, shrimp, several fish and mites, as shown in Table 1. This first analysis was performed in the in-house laboratory of the AUVA Rehabilitation Clinic in Tobelbad (Austria) as part of the initial examination (Phadia 100 from ThermoFisher, Waltham, MA, USA). After receiving the results listed in Table 1, additional allergen components were analyzed through ImmunoCAP at the Clinic for Dermatology and Venerology, Medical University Graz. Moreover, a prick-to-prick test was performed on the patient’s forearm. In addition to the positive control with histamine and the negative control with 0.9% saline solution, an unprocessed raw piece of squid was tested. The squid was purchased in an Austrian grocery store, the exact species is not known. 

Subsequently, samples with patient serum were sent to the “Luxembourg Institute of Health, Department of Infection and Immunity” to perform additional established tests: protein in a concentration of 4.2 mg/mL was extracted from squid of the species “Illex argentinus”, which is frequently available and consumed in Europe, centrifuged and subjected to heat treatment, resulting in an extract of thermostable protein (concentration 3.4 mg/mL). Both the native and the heated squid extract were applied to a gel electrophoresis (SDS-PAGE) and stained (Coomassie Blue stain). Both the native and heated squid extract were subsequently tested in immunoblot. A commercially available anti-tropomyosin antibody (Anti-Shrimps-Tropomyosin AK, diluted 1 : 10,000 ref. PA-SHM from Indoor Biotechnologies, Cardiff, UK) and patient serum (diluted 1 : 3) were used. In addition, three negative controls were performed: only buffer solution, a pool of IgE-negative sera, and a pool of IgE-positive sera (mostly from birch pollen-allergic patients) were used. 

Squid tropomyosin was isolated from I. argentinus using ion exchange chromatography (total amount of 1.25 mg purified tropomyosin from 10 mg total extract). Specific IgE binding using patient serum versus allergenic protein preparations was quantified based on an ELISA test. Patient serum was also tested against shrimp extract (prepared at the Luxembourg Institute of Health, Department of Infection and Immunity) and Pen m 1 (tropomyosin from shrimp Penaeus monodon). Only native extracts (not heated) were used in the ELISA. In addition, cross-inhibitions with squid tropomyosin and shrimp tropomyosin were performed. 

## Results 

The results of the in-house ImmunoCAP analysis are shown in Table 1. While total IgE level was not elevated, specific IgE antibody levels against squid and shrimp were significantly increased (CAP class 4 each). The specific IgE antibody level against the storage mite Acarus siro was increased in CAP class 4 too. The specific IgE levels against the two house dust mites Dermatophages pteronyssinus and Dermatophages farinae were slightly less elevated (CAP class 3 each). The specific IgE levels against fish were not increased, in accordance with the patient’s medical history. The results of the additional ImmunoCAP analysis at the Department of Dermatology and Venerology, Medical University Graz, are shown in Table 2. The total IgE level was concordantly not elevated. The specific IgE antibody levels against squid and shrimp were slightly less elevated than in the preliminary in-house ImmunoCAP (CAP class 3). The specific IgE antibody levels against the mites Acarus siro and Dermatophages pteronyssinus were also elevated (CAP class 3). 

Component-based diagnostics show an increase in specific IgE against tropomyosin from shrimp (rPen a 1) and tropomyosin from house dust mites (rDer p 10). The analysis of specific IgE antibody levels against tropomyosin from squid is not commercially available. Specific IgE antibody levels against the major allergens from the feces and body of the house dust mite (rDer p 1 and rDer p 2) were not elevated. 

**Figure 1.**
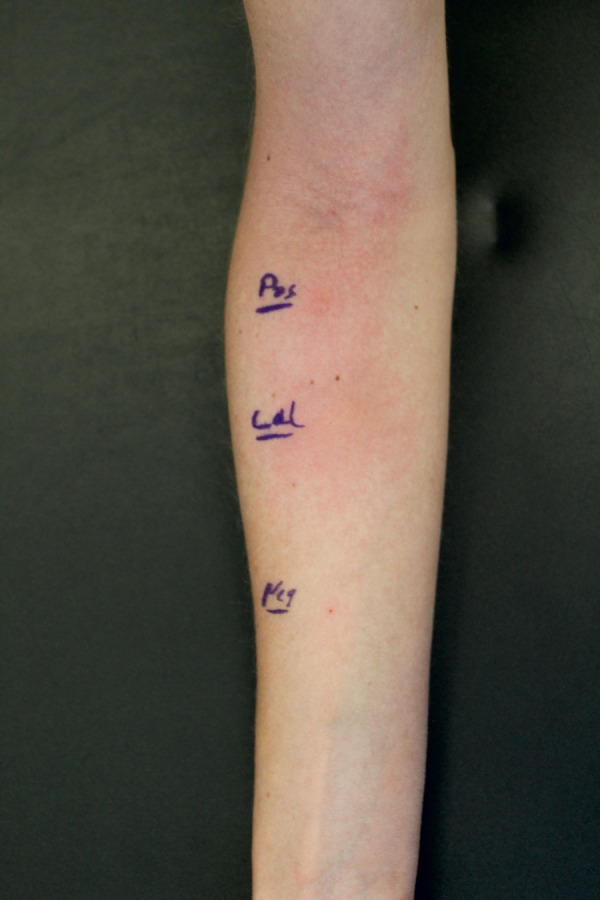
Forearm of the patient, seen ~ 20 minutes after prick-to-prick testing. The prick-to-prick site with squid is located in the middle of the patient’s forearm and is marked “Cal” (for calamari).

By prick-to-prick testing, a ++ positive reaction to the tested squid occurred as shown in Figure 1. The positive control was also ++ positive, the negative control showed a negative result. 

For further clarification of the suspected allergic reaction to tropomyosin from squid, the additional tests described in “materials and methods” were carried out at the “Luxembourg Institute of Health, Department of Infection and Immunity”. After application of the native and heated protein extracts from the squid Illex argentinus on gel electrophoresis (SDS-PAGE) and staining (Coomassie Blue stain), a dominant tropomyosin-like band was found at ~ 35 kDa. Smaller bands were observed in the heated extract, while thermostable tropomyosin was preserved [13]. In the immunoblot, tropomyosin from the native and the heated squid extract was detected by both the commercially available antibody and the patient serum and was visible as one band. In the ELISA, the highest IgE antibody level was found against squid extract (21.5 kUA/L), followed by IgE antibody levels against squid tropomyosin (11.3 kUA/L) and shrimp extract (10.9 kUA/L). These are mean values, as two replicates were analyzed in each case. After cross-inhibition, squid tropomyosin was found to be a potent inhibitor. It inhibited the IgE binding on shrimp tropomyosin by up to 85%. When shrimp tropomyosin was used as an inhibitor, the inhibition was less strong (~ 60%). 

## Discussion 

Allergic reactions to seafood are well known in the medical literature. Tropomyosin was described as a triggering allergen from shrimps as early as 1981 [9]. The vast majority of allergic reactions to seafood are caused by crustaceans [10]. Although allergic reactions to squid are much rarer, they have also been known for many years. Tropomyosin from the squid Todarodes pacificus (other species of the same family as the squid Illex argentinus used in the current case) was first described as an allergen in 1996 [15]. For the patient presented who has been working as a cook’s mate and suffering from respiratory distress when cooking squid, the fact that tropomyosin is a protein that does not lose its allergenic potential when heated is relevant. It has even been discussed that heating may increase antibody reactivity to tropomyosin [6, 10, 14]. Based on the repeated statements of the patient that skin lesions occurred exclusively after contact with squid, a cutaneous type I reaction after contact with squid was suspected. However, with symptoms dating back several years and never having consulted a physician, there was no evidence for this suspicion. A verifiable urticarial reaction to cutaneous contact with squid occurred in the prick-to-prick test. The prick-to-prick test is an easy, quick to perform and very practical test by which the occurrence of skin lesions could be objectified. This is of particular relevance since no corresponding commercial test extract is available. 

In the ImmunoCAP, increased levels in the extract-based analysis for specific IgE antibodies against squid, shrimp, and mites were found. The IgE antibody levels against tropomyosins from shrimps and house dust mites were also elevated. The low total IgE level and the lack of evidence of atopic diathesis in the patient additionally indicate a clinical relevance of the elevated levels. Analysis of specific IgE against tropomyosin from squid using ImmunoCAP is not commercially available. However, a reaction to tropomyosin extracted from squid and an increased IgE antibody level against extracted squid tropomyosin could be detected by immunoblot and ELISA at the Luxembourg Institute of Health, Department of Infection and Immunity. Furthermore, increased IgE antibody levels against shrimp extracts could be shown. 

A high cross-reactivity between tropomyosins from squid, shrimps, mites, insects, and other invertebrates is known. This is mainly due to the high structural similarity of tropomyosins from invertebrates. The sequence homology of tropomyosins of shrimps and squids is ~ 62%, for shrimps and mites it is even 81% [16]. 

The question arises whether the primary sensitization was actually to squid or whether sensitization to house dust mites was present first and the reaction to squid can be interpreted as a cross-reaction. Due to the high prevalence of sensitization to house dust mites and the high cross-reactivity between tropomyosin from house dust mites and seafood, it would be reasonable to assume that exposure to house dust mites caused the primary sensitization to tropomyosin and the reaction to seafood is a cross-reaction [6, 17]. This has also clearly been shown in earlier studies: specific IgE antibodies against shrimps could be detected in groups of people sensitized to house dust mites without any exposure to seafood (for example, Orthodox Jews) [18]. 

In the patient presented here, however, symptoms occurred for the first time during squid processing. These showed an acute onset and healed quickly after the end of the exposure to squid. Since the change of profession, the patient is permanently free of symptoms. The patient explicitly negates any symptoms from contact with shrimps or other crustaceans. She did not show any typical symptoms of an allergy to house dust mites either in childhood and youth or before, during or after her occupation as a cook’s mate. The specific IgE antibodies against allergen extracts were found more than twice as high for squid as for house dust mites. In addition, the specific IgE antibodies against the most relevant major allergens from the feces and body of house dust mite (rDer p 1 and rDer p 2) were negative. In Europe, however, over 95% of all mite allergy patients are sensitized to Der p 1 and Der p 2 [17]. This points against a primary sensitization to house dust mites. Due to the clearly increased level of the specific IgE antibody against the tropomyosin of house dust mite (rDer p 10), the authors assume a cross-reaction to house dust mites with primary, cutaneous sensitization to tropomyosin from squid. In addition, in the analysis of cross-inhibition, squid tropomyosin turned out to be a potent inhibitor, whereas in the reverse experiment, the inhibition by shrimp tropomyosin was less strong. This also speaks for a primary sensitization to squid. Despite the significantly increased specific IgE antibodies against the mite Acarus siro, the authors also assume a cross-reaction to tropomyosin, since the patient did not describe any symptoms of an allergy to mites. 

The possibility of cross-reactions is discussed in literature both after primary mite sensitization and after primary seafood sensitization. Due to the high similarity of the amino acid sequence of tropomyosins of different invertebrates, cross-reactions are well known in in vitro diagnostics. Primary sensitization can therefore be induced by seafood, mites, or other invertebrates. A dependence on individual factors of the immune system as well as on the route of sensitization and the amount of exposure is discussed. The cross-reactions found may, but do not necessarily have to, be clinically relevant and cause corresponding symptoms on exposure [19]. The authors of a Spanish study looking at patients sensitized to mites and seafood came to a similar conclusion: The vast majority of the group is assumed to be primarily sensitized to mites. In a smaller group of patients who only reacted to tropomyosin from mites and seafood, but not to Der p 1 and Der p 2, the authors assume a primary sensitization to tropomyosin from seafood [20]. 

In the here-presented patient, the increased specific IgE antibody levels against shrimp and tropomyosin from shrimp (Pen a 1) are also considered a cross-reaction. 

Based on the results of the in vitro and in vivo tests as well as the clearly matching patient history, the recognition of an occupational disease was granted. A pneumological examination was performed at the same time. Exogenous-allergic bronchial asthma due to the type I allergy to squid was diagnosed and was also recognized as an occupational disease. 

## Funding 

None. 

## Conflict of interest 

The authors state that there are no conflicts of interest. 

**Table 1.** ImmunoCAP testing in the AUVA Rehabilitation Clinic Tobelbad.

	kU/L	CAP class
Total IgE	56.0	
*Dermatophag. pteronyss.*	8.2	3
*Dermatophag. farinae*	9.6	3
*Acarus siro*	20.3	4
Squid	18.8	4
Shrimp	22.8	4
Salmon	0.1	0
Tuna	0.2	0
Cod	0.1	0

**Table 2.** ImmunoCAP testing in the Department of Dermatology and Venerology, Medical University Graz.

	kU/L	CAP class
Total IgE	35.4	
*Dermatophag. pteronyss.*	4.6	3
rDer p 1	0.0	0
rDer p 2	0.0	0
rDer p 10	11.6	3
*Acarus siro*	11.4	3
Squid	10.5	3
Shrimp	13.0	3
rPen a 1 tropomyosin	10.1	3
